# Causes of the Low Rate of Mortality among Foreign Residents at the Sandwich Islands

**Published:** 1878-07

**Authors:** Henry M. Lyman

**Affiliations:** Professor of Physiology and of Nervous Diseases, Push Medical College, Chicago


### CAUSES OF THE LOW RATE OF MORTALITY AMONG THE FOREIGN RESIDENTS AT THE SANDWICH ISLANDS.

By Henry M. Lyman, M. D.,

*(Professor of Physiology and of Nervous Diseases, Rush Medical College, Chicago.)*

The remarkable paper by Judge Caton in the June number of the Journal and Examiner regarding the low rate of mortality which obtains among the foreign inhabitants of the Sandwich Islands, while the native inhabitants are rapidly diminishing in number under the influence of epidemic diseases and the untoward circumstances of a sudden change from the savage state to comparative civilization, affords interesting evidence in corroboration of the doctrines enunciated by Herbert Spencer in his "Principles of Biology." Spencer shows that the natural tendency of things is in the direction of an equilibrium of forces. Every organized body is the result of an equilibrium between internal forces and external forces. Every living body is the result of the establishment and preservation of an equilibrium between its internal vital forces and the external, incident forces of nature which tend to disintegrate every existing form. Now, when the incident forces, whether physical, spiritual, or both—it makes no difference which—are numerous and complicated, a corresponding multiplicity and complexity of the internal vital adjustments will be necessary, in order to effect the preservation of a living equilibrium. The result will be a highly organized and enduring structure. This is precisely the result which experience discovers. In the temperate zones of the earth the number and complexity of the conditions of existence is the greatest. It is here that the greatest changes and varieties of temperature and climate are experienced. It is here that, consequently, the greatest variety of occupation and mode of life is possible, so that it is in precisely this portion of the earth that we find that complexity and perfection of physical structure, *growing out* of perfect equilibrium of forces, which gives special toughness and power of endurance to that portion of the human species which inhabits the favored zone.

There is, however, another consequence of the natural tendencies towards equilibrium which is not so conspicuous, and is therefore often overlooked by medical writers. When the incident forces have operated with great uniformity for a succession of generations upon a given race of beings, there becomes established such a perfection of equilibrium between vital forces on the one hand and the antagonistic forces on the other, that a new element of danger is evolved. Exposed to a uniform succession of similar incidences, the living organism becomes exceedingly strong in its power of resisting the impact of that particular group of forces; but, experiencing no need of adjustment against other kinds of incident force, it becomes less and less complex in its structure, and correspondingly less capable of resisting the shock of new and unexpected modes of motion. This lower form of organization is found in those regions where the conditions of life are characterized by great uniformity. It is reduced to its lowest terms of simplicity, and consequent lack of adaptability to various and sudden changes, in those parts of the earth where the temperature, climate and mode of life are the most uniform, and where the species is most isolated. These are the conditions which have made the Pacific islanders what they are; hence their inferior power of resistance when exposed to new influences that are opposed to life.

Such is the Spencerian mode of stating the case. Philosophy aside, we recognize the same facts every day, and much of our practice is based upon a semi-conscious recognition of these facts. Take, for example, a family of young children among the wealthier classes in society. Surround them with every luxury, in a winter-palace, with a constant temperature of 72° F., and they will for a time enjoy excellent health. They are like a tribe of Polynesians shut up on a little island in the middle of the Pacific. But the little things, though they never have measles or scarlet fever, or any other horrid infectious disease, become surprisingly delicate and tender. A little too much dinner makes them yell with colic; a new tooth produces violent convulsions; and a ride in the open air would fairly snuffle them out of existence. They have become thoroughly equilibrated with a certain limited number of incident forces; and this perfection of adjustment to those narrow conditions leaves them preternaturally sensitive to every novel influence. They may be likened to a fortification in which all the labor has been concentrated upon the sea-ward side—it is impregnable against a naval attack, but wholly defenceless against an approach from the land.

Now when we desire to "harden" a delicate child, we proceed to expose him to the greatest possible variety of conditions—incident forces, as Spencer calls them. We turn him out of doors every day; we take off some of his extra wraps; we bathe him with cool water; we make him practice all kinds of games; we get him vaccinated; we let him run the risk of infection, and are glad when he has done with the measles and scarlet-fever and whooping-cough; we do not fear the changes of season, but rather welcome them as agents in the great process of building a man. If the little fellow can't stand the operation, sensible people leave to sentimentalists the task of weeping over the unhappy "victim" of exposure, and renew the experiment with the next subject which opportunity affords. It is by precisely such processes of natural selection that the earth has become peopled with just such races as now exist. When the conditions which have produced such results are anywhere suddenly modified, definite results may always be expected. If the simple mode of life of a Polynesian tribe be suddenly changed in the direction of greater complexity, it will inevitably cause a fearful mortality from diseases against which their section of the human species has never had occasion to adjust itself. But if a portion of a highly organized race, like the Anglo-Saxon, for example, be transplanted to a region where the conditions of life are less complex, but not too far removed from those to which it has always been accustomed, the first consequence will be greater immunity from disease and a diminished mortality. This is owing to the hereditary transmission of the ancestral complexity of structure, which for a time preserves those adjustments of the organism by which it is enabled to contend successfully against adverse forces. In time, however, the universal tendency to perfect equilibrium between internal and external forces will assert itself, and the descendants of the most highly organized races, especially if shut out from intermarriage and intercourse with more favored branches of the parent stock, will sink to the level of the inferior variety.

The bearing of all this upon the course of epidemic disease in any given community is obvious. Exclude measles, for example from an island for a century or more. A race of men is produced whose physical organization has never been disturbed by the impact of the causes of measles. The consequence is a structure wholly out of equilibrium with such a force, and exceedingly sensitive to its influence. Now introduce the disease into such a community, and it will spread like wild-fire, with a terible severity and great mortality. Witness the epidemics of measles at the Faroe Islands, and at the Sandwich Islands. At length, however, the organization of the race adjusts itself to the new force; it is no longer sensitive; the epidemic "dies out" and if soon reintroduced its lack of fatality is like that observed where the disease is always endemic. We see the same thing illustrated by the course of scarlet-fever in our own communities. This is, also, the reason why the first outbreak of an epidemic, like cholera, is attended with a rate of mortality which steadily diminishes with the progress of the pestilence. These considerations, also, explain the futility of all those efforts at stamping out disease which are based simply upon non-intercourse. Disinfection and such processes as vaccination, afford the only methods which afford a reasonable hope of limiting the ravages of communicable disease.

